# Perceptions and practices of pharmaceutical wholesalers surrounding counterfeit medicines in a developing country: a baseline survey

**DOI:** 10.1186/1472-6963-11-306

**Published:** 2011-11-11

**Authors:** Mohiuddin H Khan, Manabu Akazawa, Eav Dararath, Heng B Kiet, Tey Sovannarith, Nam Nivanna, Naoko Yoshida, Kazuko Kimura

**Affiliations:** 1 Drug Management and Policy, Institute of Medical, Pharmaceutical and Health Sciences, Kanazawa University, Kakuma-machi, Kanazawa City, 920-1192, Japan; 2 Department of Public Health and Epidemiology, Meiji Pharmaceutical University, 2-522-1 Noshio, Kiyose, Tokyo, 204-8588, Japan; 3 Department of Drugs and Food, Ministry of Health, 8 Ung Pokun Street, Mittapheap, Khan 7 Makara, Phnom Penh, Cambodia; 4 National Health Product Quality Control Center, Ministry of Health, 151-153 Kampuchea Krom Bldv., Khan 7 Makara, Phnom Penh, Cambodia

## Abstract

**Background:**

Recent investigations by the Ministry of Health of Cambodia suggest that counterfeit medicines have been introduced into the pharmaceutical market in tampered packaging. To further explore this possibility, an interview survey was conducted at the wholesaler level to investigate the medicinal supply chain in Cambodia.

**Methods:**

Managing executives of 62 (83.8%) registered wholesalers of modern medicines in Cambodia were interviewed in 2009 on their knowledge of, perception on, and practices related to counterfeiting issues through a semi-structured questionnaire.

**Results:**

According to our findings, 12.9% of the wholesalers had encountered counterfeit medicine. However, they demonstrated a variety of perceptions regarding this issue. A majority (59.7%) defined counterfeit medicines as medicines without registration, while other definitions included medicines that were fraudulently manufactured, medicines without a batch/lot number, those containing harmful ingredients or a reduced amount of active ingredients, and expired medicines. Additionally, 8.1% responded that they did not know what counterfeit medicines were.

During procurement, 66.1% of the wholesalers consider whether the product is registered in Cambodia, while 64.5% consider the credibility and quality of the products and 61.3% consider the reputation of the manufacturers. When receiving a consignment, 80.6% of wholesalers check the intactness of medicines, 72.6% check the specification and amount of medicines, 71% check Cambodian registration, 56.5% check that the packaging is intact, 54.8% check batch and lot numbers, 48.4% check the dates of manufacture and expiration, and 9.7% check analytical certificates.

Out of 62 wholesalers, 14.5% had received medicines that arrived without packages or were separated from their packaging and had to be repacked before distribution. Significant statistical association was found between wholesalers who received medicines separately from their packs/containers and who consider their belief on reliability of pharmaceutical products of certain manufacturing country during procurement (Chi-square: 12.951, *P *= 0.002). When wholesalers divide medicines from larger packs into smaller ones, 54.8% use packaging purchased from local markets.

**Conclusion:**

A number of wholesalers think counterfeit medicines are medicines without registration, and/or do not have any uniform ideas on the issue and what to do, when they find or suspect counterfeits. Furthermore, their strict adherence to anti-counterfeiting measures is urgently needed.

## Background

Like many other public health problems, the counterfeiting of medicine is an important issue that should receive careful attention in developing countries. In Cambodia, the Ministry of Health (MoH) and law enforcing agencies first became aware of the issue in the late 1990s, and since then, the government in cooperation with several international organizations (such as World Health Organization (WHO), INTERPOL, USAID, US Pharmacopeial Convention (USP), Japan Pharmaceutical Manufacturers Association (JPMA) etc.) has been struggling to stamp out the problem and to prevent further damage to communities [1-7]. However, the point at which to start tackling this problem is still unclear in many parts of the world, as well as in Cambodia [8].

Invariably, after manufacture, medicinal products are transferred through several hands to reach their customers. Even in developed countries, distributors are identified as the most critical link in the legitimate pharmaceutical supply chain and the point of entry for most counterfeit medicines [8,9]. Counterfeit medicines have been discovered at various points in the supply chain: at the industry level, at the wholesale level, the packaging level, and so forth [10,11]. In the developing world, the situation could be even worse, especially where regulations are weakly enforced [12].

According to the MoH, Cambodia, counterfeit medicine is one, which is deliberately produced with incorrect or wrong active ingredients or without active ingredients or unregistered, in which, amounts of active ingredients are deliberately outside the defined pharmacopoeial standard or which is deliberately and fraudulently mislabelled with respect to the identity source or with fake packaging, or repacked or produced by unauthorized person [2]. In 2001 and 2004, the MoH, Cambodia, in cooperation with WHO reported the prevalence of 10.43% and 21.13% counterfeit medicines respectively among some essential medicines (such as antimalarials, analgesics, antibiotics, vitamins and steroids) in the country [2,13]. A study conducted during 2002-2003, reported 92.7% substandard aspirin in private outlets [14]. In 2006, another study found 58% counterfeit and substandard antimalarials in licensed and 75% in non-licensed outlets [15]. In many cases, the counterfeit products identified in retail shops were in open packages and/or were unregistered [7,16]. Some of the counterfeit medicines were identified as two or more physical types of medicines and active ingredients were mixed together in one container or box [7]. These discoveries prompted the Cambodian Ministry of Health to look into the pharmaceutical supply chain, emphasising issues of counterfeiting. In this connection, the Department of Drugs and Food (DDF), MoH, Cambodia conducted a face-to-face interview survey among pharmaceutical wholesalers in collaboration with Kanazawa University, Japan, with the objective of assessing wholesalers' sensitisation to counterfeit issues and observing their compliance with DDF's guidelines. It might be worth mentioning that DDF is the medicine regulatory authority in-charge of monitoring the pharmaceutical sector in Cambodia.

## Methods

This cross-sectional survey was conducted in July 2009 on signing an agreement between Ministry of Health, Cambodia and Japan Pharmaceutical Manufacturers Association (JPMA) and after an ethical clearance approval of Kanazawa University, Japan. Advice was also sought in this regard from local Cambodian institutions. A list of private sector wholesalers was prepared beforehand in consultation with the DDF. All of the wholesalers in the list were contacted for face-to-face interviews, excluding those who might have changed addresses or closed down their businesses. Based on their addresses in different locations of the capital city, Phnom Penh, the list was then distributed to two different teams of interviewers. Each team consisted of a Japanese research investigator and two locally recruited Cambodian members.

A semi-structured questionnaire was prepared (based on a questionnaire survey conducted in 2008 at retail pharmacy level in Cambodia) for the interview, focusing on wholesalers' practices and perceptions surrounding counterfeit medicine issues, which could be categorised as questions related to the following: 1) attributes of the respondent and wholesale business, 2) perception and practices related to procurement and storage of medicines, 3) perceptions and practices related to medicine quality, and 4) observation on handling medicines in the ware house [17]. The questionnaire was translated into the Khmer language for Cambodian respondents. The questionnaire was then discussed with the Japanese and local researchers, and staff of Ministry of Health.

All participants in the interview teams were provided with training to avoid team bias and were instructed that the interviewers should not provide leading questions during interviews. The questionnaire was then pretested among different wholesalers in Phnom Penh and edited before starting the final survey. Interview teams contacted managing executives of wholesale businesses. Interviewers explained the reason for the study to the respondents and requested their cooperation. A small gift was given as a token of appreciation to each interviewee, and informed consent was obtained before proceeding.

The respondents were encouraged to reply honestly and without fear. Interviewers explained to respondents that there was no threat of future action based on their responses and that all information would be handled maintaining utmost confidentiality in accordance with the medical ethics committee of Kanazawa University. Interviews were conducted in Khmer language, however, in some cases, when respondents were foreigners, interviews were taken in English.

Multiple answers were accepted for some of the questions. For the observational section of the questionnaire, interviewers visited the warehouses of the wholesalers after obtaining respondents' permission. The interviewers checked whether or not medicines were kept well arranged according to medicine types and/or classification; cleanliness, tidiness and sufficiency of space in the stores were assessed subjectively on agreement of the members of the interview team. The physical properties, such as size, shape, colour, printed information, emboss, hologram etc. of three or four samples of medicines from the shelves in the warehouses were examined to find out presence of two or more types of medicines mixed together in one container or box, presence of expired medicines and medicines without registration labels. Each of the filled-out questionnaires was given a specific interview number immediately following its completion.

Data analysis was performed using SPSS release 17.0.0 (Chicago, SPSS Inc.). Where appropriate, Fisher's exact test was performed to identify whether there was any significant relation among the variables of perceptions and practices. However, only relevant significant associations are mentioned in the results section. Statistical significance was set at the level of 5%.

## Results

A total of 62 wholesalers were interviewed. The mean age of the respondents was 38 years (mode: 36 yrs; SD: 10.89) and the majority (50.81%) of them were male. Among those interviewed, 35% were company owners; 48% were directors, managers, or administrative heads; 8% were general staff; and 8% held other positions. Their educational background varied; 27% of the respondents were pharmacists and 8% were doctors, whereas 21% had some sort of business degree and the rest (44%) were from other educational backgrounds. A large number (40%) of the wholesalers had a staff of fewer than ten people, and 56% had been operating their businesses for less than five years in Cambodia.

### Suppliers and customers

When the wholesalers were asked about the primary sources of their products, 95.2% (n = 59) of them responded that they procure their products directly from foreign manufacturers, whereas 12.9% (n = 8) of them procure them through an importing agent; 1.6% (n = 1) of them obtain products from another distributor or supplier, and 6.5% (n = 4) of them procure products from domestic manufacturers. The majority (96.8%, n = 60) of the wholesalers said that their customers were pharmacies (i.e.: legal outlets, supposed to be run by pharmacists) and Depot A (i.e.: legal outlets, supposed to be run by assistant pharmacists, those who received three years of pharmaceutical training) and Depot B (i.e.: legal outlets, supposed to be run by retired doctors or nurses) types of outlets. Additionally, 80.6%, (n = 50) of them distribute medicines to private clinics and practitioners. However, 8.1% (n = 5) of them supply products to any drug stores regardless of license, and 4.8% (n = 3) of them sell to another wholesaler. The number of medicinal products that they were dealing with varied from 3 to 450 (mean: 80.94; SD: 98.18).

Sixty (96.7%) of the wholesalers always procure their medicines from the same manufacturer(s)/company/companies, mainly due to their trust (73.3%, n = 44) of these companies and the quality of products (71.7%, n = 43); 18.3%, (n = 11) procure their medicines from the same supplier due to reasonable price, and 6.7% (n = 4) of them do so because of supplier can provide a wide variety of medicines. Occasionally, the wholesalers had refused to purchase medicines from some suppliers/sources; their most common explanation for this was that the suppliers were not trustworthy (55.6%, n = 25) in their business dealings. Similarly, when they refused to supply to a customer, it was most often the customers' trustworthiness that made them to do so (59.1%, n = 13).

### Procurement

**Figure 1:**
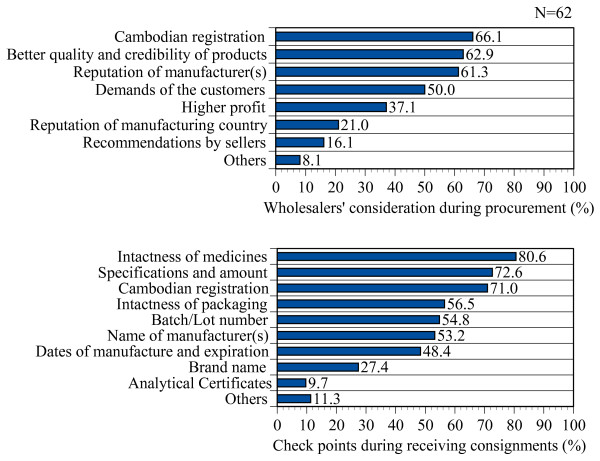
**Wholesalers' considerations and checkpoints during procurement**.

In response to the question regarding their considerations when procuring medicine(s), a majority of wholesalers (66.1%, n = 41) reported that they consider Cambodian registration, while 62.9% (n = 39) of them consider the quality and credibility of the products; 61.3% (n = 38) of them consider the reputation of the manufacturer, 50% (n = 31) consider demands of customers, 37.1% (n = 23) consider profits, 21% (n = 13) consider the reputation of the manufacturing country, and 16.1% (n = 10) consider the recommendations of the retailers (sellers). However, when receiving a consignment, 80.6% (n = 50) of respondents check any damage of the medicines due to shipment or handling and make sure of their intactness, 72.6% (n = 45) of them check the specification and amount, 71% (n = 44) check Cambodian registration, 56.5% (n = 35) check the intactness and any damage of packaging, 54.8% (n = 34) check the batch and lot number, 53.2% (n = 33) check the name of the manufacturer, 48.4% (n = 30) check the dates of manufacture and expiration, 27.4% (n = 17) check the brand name, and 9.7% (n = 6) of them check the analytical authorisation of the products (Figure 1). In principle, all pharmaceutical consignments required to be accompanied with certificates of analysis (COA) issued by the manufacturers, which should be based on the results obtained from the analysis of the lots/batches that are being shipped [18]. Those who consider registration or check registration in the consignments were asked about their reasons for doing so. In reply, 81.6% (n = 40) of them reported that they were following the instruction of authorities, while 67.3% (n = 33) of them said that they do so because of their belief that registration guarantees quality, and 8.2% (n = 4) of them ascribed the practice to their experience of low quality in unregistered medicines.

### Packaging practice

In response to a question, 14.7% (n = 9) of the wholesalers reported that they had received medicines separately from their packaging or with missing packaging. A significant statistical association was found between wholesalers who received medicines outside of their packaging and those who consider the reputation of the manufacturing country during procurement (Chi-square: 12.951, *P *= 0.002)

**Figure 2:**
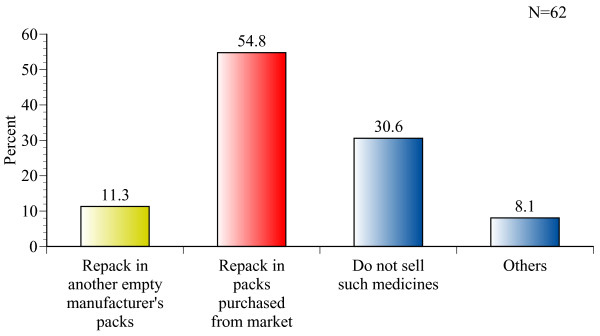
**How do wholesalers repack medicines when they need to divide large packs?**.

In situations where wholesalers needed to divide medicines from large packs or containers in order to supply a portion of the medicine to a customer, 54.8% (n = 34) of them repack medicines in ordinary, commercially purchased paper or plastic packaging. By comparison, 11.3% (n = 7) of the respondents repack medicine in another empty container belonging to the same medicine or same manufacturer, and 30.6% (n = 19) do not sell medicines in such cases (Figure 2).

**Figure 3:**
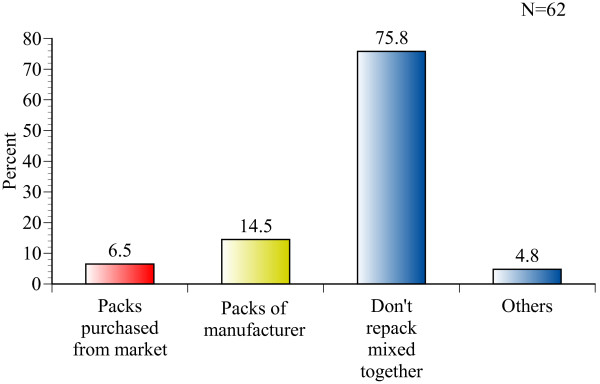
**How do wholesalers repack two or more kinds of medicines?**.

In another question, wholesalers were asked what they usually do if they need to repack two or more kinds of medicines that treat the same disease or bear the same active ingredient in cases where they will be sold to a single customer, provided that there are not enough packs or containers available. Four (6.5%) respondents replied that they re-pack medicines in packaging purchased from markets, 9 (14.5%) said they use another container for the same medicine, and 47 (75.8%) said they always supply medicines only in genuine containers or packs (Figure 3). Our findings also suggest that medicines are sometimes mixed in the same package. Detailed observational findings are summarised in Table 1.

**Table 1:** Results of observation of storage practice

	Observation	Yes
1	Medicines arranged in the shelves according to different classification, and not mixed together (n = 52)	38 (73.1%)
2	Medicines are stored maintaining tidiness and cleanliness (n = 51)	43 (84.3%)
3	Is there sufficient space in the store? (n = 52)	44 (84.6%)
4	Does the wholesaler maintain stock records for different kinds of medicines? (n = 52)	48 (92.3%)
5	Are there medicines mixed together with other types of medicines in a container or box? (n = 51)	6 (11.8%)
6	Presence of expired medicine on the shelves with other medicines (n = 52)	0
7	Presence of medicines without registration labels mixed with other medicines with registration on the shelves (n = 52)	1 (1.9%)

### Counterfeit medicine issue

**Figure 4:**
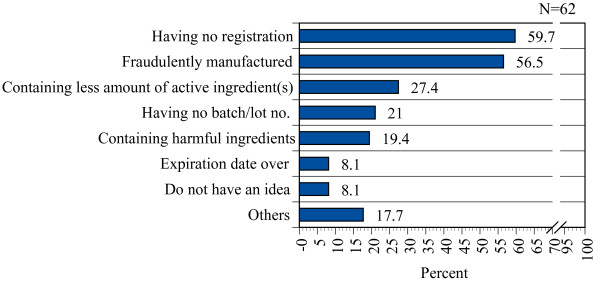
**Wholesalers' conceptions of counterfeit medicines**.

Eight (13.3% of 60) of the respondents said that they had previously seen counterfeit medicines and they identified those from their printed lebels, addresses or source, feed back from customers, different design/characteristics of package, logo and having no registration number. When they were asked about their perceptions of counterfeit medicines, a majority (59.7%, n = 37) of respondents expressed the opinion that counterfeit medicines are medicines having no registration and/or were fraudulently manufactured (56.5%, n = 35) (Figure 4). However, at least eight (12.9%) of them did not have any clear conception of the meaning of the term. Five of them replied that they did not know what counterfeit medicines were, and three others replied that counterfeit medicines are medicines with reduced amounts of active ingredients, cheaper medicines, or expired medicines.

According to the wholesalers' statements, if they received any counterfeit medicine, 72.4% (n = 42) of them would report such a case to the regulatory authorities, and 27.6% (n = 16) of them would either send the medicine back to its source or separate and discard the medicine without informing the authorities.

To assess wholesalers' awareness of regulations, respondents were asked whether they were aware that they must inform DDF before dispatching any consignment, and it was found that 35% (n = 21) of them did not know this requirement. A statistically significant association was found between wholesalers who were not aware of the requirement and those who do not consider Cambodian registration during procurement (Chi-square: 4.29, P = 0.05).

## Discussion

Most often, pharmaceutical products find their way from manufacturers to pharmacies through wholesalers. However, there are cases where the wholesalers sell medicines to secondary or tertiary wholesalers or distributors [19-21]. The findings of this survey suggest that wholesalers procure their products from different sources, suppliers and distributors and that these multiple actors in the supply chain may potentially increase the risk of intrusion of counterfeit products if regulation is limited. The survey was limited in that it was not conducted by professional interviewers, where, two teams were deployed for the interviews and interviews were conducted in both Khmer and English languages; as such, it was not possible to completely rule out the possibility of interviewer bias. Nevertheless, to minimise such bias, training and feedback discussions were held after the interviews. Another limitation of the survey is that it was designed on certain background of counterfeit findings of the country and may not cover comprehensively wholesalers' perception and practices on dealing with medicines. We believe that the results at least represent baseline information on Cambodian wholesalers and could help Cambodian regulators take future steps.

This survey has shown that cooperation and trust among different stakeholders in the supply chain might play important roles in their partnership and in their efforts to maintain quality. It is encouraging that most of the wholesalers consider Cambodian registration during procurement when receiving consignments because medicines without registration were significantly associated with counterfeit medicines in an earlier study [16]. Nevertheless, only a few respondents noted that they check analytical certificates.

Some of the wholesalers confirmed that they sometimes receive medicines separately from their packaging and that they repack them before resale. This finding was also associated with wholesalers' consideration of the reputations of the manufacturing countries during procurement. This might be important information because package condition and foreign origin were previously identified as other influential factors for counterfeit medicines [16]. Moreover, our survey findings suggest that some of the wholesalers repack medicines by procuring extra containers from local markets, although DDF does not allow wholesalers to import or procure medicines without packages, nor are they allowed to repack medicines. It may happen that due to wholesalers trust and believe on products of some of the manufacturing countries, they slip from checking or over look the packaging requirements during receiving medicinal consignments. If packing or repacking at the wholesaler level is unavoidable due to the convenience of transportation and/or distribution, we suggest existing regulation should be reinforced to require proper labelling to protect genuine medicines.

Some of the respondents in this survey did not have a clear conception of counterfeit medicines and were unaware of what they should do if they suspect any counterfeits. In these situations, there is a strong need to orient and sensitise wholesalers to issues of counterfeit medicine. Anti-counterfeit measures need to be incorporated into a set of guidelines to be developed for wholesalers [22]. This survey shows that wholesalers' businesses are licensed and regulated by a number of authorities, which makes them difficult to monitor [19]. The association found among wholesalers who were unaware of up-to-date regulatory information and those do not consider Cambodian registration during procurement may signify that some information gaps exist among stakeholders. To strengthen the regulatory system, information-sharing components in the form of advocacy workshops or meetings should be arranged on a regular basis.

## Conclusion

A major proportion of wholesalers are not properly informed on issues of counterfeit medicine and how to handle such cases. To protect the supply chain of pharmaceutical products from the intrusion of counterfeit medicines, distributors and wholesalers should be oriented and sensitised to countermeasures against counterfeit medicines.

## Competing interests

The authors declare that they have no competing interests.

## Authors' contributions

MHK, KK, ED, HBK, TS and NY participated in the designing, field activities and documentation of the survey; MHK, ED, BHK, TS, NN and NY participated in data analysis; MHK, MA, ED, TS, NN, NY and KK participated in the interpretation of results. MHK wrote the first draft of the manuscript. All the authors participated in the critical review of the draft manuscript, editing, and finally approved its submitted version.

## Pre-publication history

The pre-publication history for this paper can be accessed here:

http://www.biomedcentral.com/1472-6963/11/306/prepub
